# Clinical characteristics of familial dysalbuminemic hyperthyroxinemia in Chinese patients and comparison of free thyroxine in three immunoassay methods

**DOI:** 10.3389/fendo.2023.1102777

**Published:** 2023-02-14

**Authors:** Linlin Zhao, Yingying Zhou, Fengjiao Huang, Xiaoyang He, Guili Mei, Shoujun Wang, Yanyan Zhao

**Affiliations:** ^1^ Department of Endocrinology, The First Affiliated Hospital of Zhengzhou University, Zhengzhou, Henan, China; ^2^ Department of Endocrinology, Boai County People’s Hospital, Jiaozuo, Henan, China

**Keywords:** familial dysalbuminemic hyperthyroxinemia, euthyroid hyperthyroxinemia, R218H, R218S, chemoluminescence immunoassays

## Abstract

**Objective:**

Familial dysalbuminemic hyperthyroxinemia (FDH) has not been thoroughly studied in the Chinese population to date. The clinical characteristics of FDH in Chinese patients were summarized, and the susceptibility of common free thyroxine (FT4) immunoassay methods was evaluated.

**Methods:**

The study included 16 affected patients from eight families with FDH admitted to the First Affiliated Hospital of Zhengzhou University. The published FDH patients of Chinese ethnicity were summarized. Clinical characteristics, genetic information, and thyroid function tests were analyzed. The ratio of FT4 to the upper limit of normal (FT4/ULN) in three test platforms was also compared in patients with R218H *ALB* mutation from our center.

**Results:**

The R218H *ALB* mutation was identified in seven families and the R218S in one family. The mean age of diagnosis was 38.4 ± 19.5 years. Half of the probands (4/8) were misdiagnosed as hyperthyroidism previously. The ratios of serum iodothyronine concentration to ULN in FDH patients with R218S were 8.05–9.74 for TT4, 0.68–1.28 for TT3, and 1.20–1.39 for rT3, respectively. The ratios in patients with R218H were 1.44 ± 0.15, 0.65 ± 0.14, and 0.77 ± 0.18, respectively. The FT4/ULN ratio detected using the Abbott I4000 SR platform was significantly lower than Roche Cobas e801 and Beckman UniCel Dxl 800 Access platforms (*P* < 0.05) in patients with R218H. In addition, nine Chinese families with FDH were retrieved from the literature, of which eight carried the R218H *ALB* mutation and one the R218S. The TT4/ULN of approximately 90% of patients (19/21) with R218H was 1.53 ± 0.31; the TT3/ULN of 52.4% of patients (11/21) was 1.49 ± 0.91. In the family with R218S, 45.5% of patients (5/11) underwent TT4 dilution test and the TT4/ULN was 11.70 ± 1.33 and 90.9% (10/11) received TT3 testing and the TT3/ULN was 0.39 ± 0.11.

**Conclusions:**

Two *ALB* mutations, R218S and R218H, were found in eight Chinese families with FDH in this study, and the latter may be a high-frequency mutation in this population. The serum iodothyronine concentration varies with different mutation forms. The rank order of deviation in measured *versus* reference FT4 values by different immunoassays (lowest to highest) was Abbott < Roche < Beckman in the FDH patients with R218H.

## 1 Introduction

Family dysalbmuinemic hyperthyroxinemia (FDH) is an autosomal dominant disease characterized by euthyroid hyperthyroxinemia due to a high affinity of defective albumin for iodothyronine. To date, several albumin gene (*ALB*) variants, namely, p.Arg218His (R218H), p.Arg218Pro (R218P), p.Arg218Ser (R218S), p.Arg222Ile (R222I), and p.Leu66Pro (L66P) have been reported in FDH patients (1), of which the R218H variant, with an increased affinity for thyroxine (T4), was first described by Sunthornthepvarakul et al. (2) and Petersen et al. (3) in 1994 and is the most common variant responsible for FDH (1). To the best of our knowledge, most of the studies including FDH subjects in the Chinese population have been published as case or pedigree reports to date. In the present study, eight FDH probands with the *ALB* R218H or R2181S mutation from unrelated Chinese families and the relatives with FDH were investigated. In addition, the reported studies regarding FDH patients of Chinese ethnicity were reviewed, the clinical characteristics of FDH were summarized, and the possible strategies for early diagnosis were explored.

## 2 Materials and methods

### 2.1 Patients

From January 2018 to August 2022, eight patients (four men and four women) diagnosed with FDH who visited the First Affiliated Hospital of Zhengzhou University clinic of endocrinology were retrospectively analyzed. The relatives of the top five probands were screened, and eight subjects were diagnosed with FDH based on thyroid function tests and gene detection. The probands and their relatives with FDH were investigated in the present study, and all belonged to Chinese Han ethnicity from Henan Province. The information included patients’ age, gender, clinical symptoms, laboratory examinations, and genetic information. Patients were enrolled after the approval from the Ethics Committee of the First Affiliated Hospital of Zhengzhou University, and written informed consent was obtained.

The inclusion criteria were as follows: (1) increased total T4 (TT4) based on repeated measurements and not accordingly suppressed thyroid stimulating hormone (TSH); (2) enthyoid; (3) normal levels of serum sex hormone binding globulin (SHBG) as well as albumin and throxine-binding globulin (TBG); (4) no mental disorders or acute medical illness; (5) no use of biotin supplements, heparin, amiodarone, exogenous estrogens, oral contraceptives, heroin and methadone, 5-fluorouracil, or clofibrate; and (6) the presence of *ALB* gene mutation. The exclusion criteria included the presence of pathogenic *THRβ* variants or variants of unknown significance (VUS).

### 2.2 Gene sequencing

Blood samples were obtained from the probands and their affected family members. Genomic DNA was extracted from the peripheral blood leucocytes, and the gene sequence variation of every proband was conducted using high-throughput sequencing. The target genes detected were given in detail in **Supplementary Table S1**. For screened relatives, only the presence or absence of the pathogenic variant known in the proband was searched using PCR-Sanger sequencing. The variants were classified following the recommendation of the American College of Medical Genetics and Genomics (ACMG).

### 2.3 Laboratory assays and imaging studies

All the laboratory assays were performed in our institution. Serum levels of total triiodothyronine (TT3), TT4, free T3 (FT3), free T4 (FT4), and TSH of the probands and affected family members were measured using the two-step chemoluminescence immunoassay with the Beckman platform (UniCel Dxl 800 Access, Beckman Coulter, Brea, CA, USA). TBG was measured using the chemoluminescence immunoassay (Immulite 2000, Siemens Healthineers AG, Erlangen, Germany). The serum albumin level was measured using the bromocresol green assay (Roche, Basel, Switzerland). The FT4 levels of the patients from pedigrees 1 to 5 were also detected using two other chemoluminescence immunoassays (Cobas e801, Roche, Swiss and I4000 SR, Abbott Laboratories, Chicago, IL, USA). The reference intervals used were provided by the laboratory. All the probands received pituitary magnetic resonance imaging (MRI), thyroid ultrasound, and some also underwent radioactive iodine uptake (RAIU) to exclude a TSH-secreting adenoma.

To determine the affinities of defective albumin for iodothyronine, the ratios of serum iodothyronine concentration to the upper limit of normal (ULN) were calculated. Furthermore, to compare the FT4 values from three common assays for thyroid function used in our region, the one-step method (Roche platform) and two-step methods (Beckman and Abbott platforms), the ratio of serum FT4 concentration to ULN (FT4/ULN) was determined and compared among three platforms.

### 2.4 Statistical analysis

Descriptive statistics were used to analyze the data and categorical variables were expressed as frequencies and percentages (%). The measurement data subject to normal distribution were expressed as mean ± standard deviation (SD). The differences in FT4/ULN ratios among the three assays were analyzed using paired samples *t*-test with IBM SPSS Statistics 25 software, and a *P* value of < 0.05 was considered statistically significant.

### 2.5 Literature summary

The CNKI database (https://www.cnki.net/), Wanfang Data Knowledge Service Platfrom (https://www.wanfangdata.com.cn/index.htmL), and Pubmed (https://pubmed.ncbi.nlm.nih.gov/) were searched from January 1999 to October 2022 period using the keyword “familial dysalbuminemic hyperthyroxinemia.” Eight articles were selected in which FDH patients of Chinese ethnicity were investigated. A total of 33 FDH patients from nine unrelated families were identified; one was excluded due to combination with hypothyroidism. Patients’ age, gender, chief manifestation, thyroid function test, and genetic information were collected. In addition, the ratio of serum iodothyronine concentration to ULN was calculated.

## 3 Results

### 3.1 Clinical characteristics

The present study included 16 patients (nine men and seven women) from eight families. The mean age of diagnosis was 38.4 ± 19.5 years. Among the eight probands, 50% of patients (4/8) complained of persistent or paroxysmal palpitation, and the other four patients (4/8) received the thyroid function tests as a routine health examination. All relatives with FDH from pedigrees 1 to 5 (8/8) had no symptoms. Two probands complaining of palpitation were also diagnosed with atrial fibrillation (**Table 1**).

**Table 1:** General information, parameters of thyroid function, and biochemical assays of the patients.

Pedigree no.	Patient no.	Gender	Age (years)	Genetype	TT3 (nmol/L)	TT4 (nmol/L)	rT3 (ng/ml)	FT3	FT4 (pmol/L)			TSH (μIU/ml)	Alb (g/L)	TBG (μg/mL)
									Roche	Beckman	Abbott			
1	1^acd^	M	55	R218S	1.87	1533.15^b^	1.14	6.87	99.40	158.98^b^	26.29	2.60	41.2	22.10
	2	M	31	R218S	3.50	1266.79^b^	1.32	6.63	78.74	142.66^b^	22.97	1.98	43.6	19.80
2	3^a^	M	10	R218H	2.39	200.10	0.94	6.57	31.80	41.98	19.65	2.87	42.0	21.10
	4	F	5	R218H	2.16	243.41	0.88	6.36	26.30	34.42	16.04	4.19	40.5	19.50
	5	F	33	R218H	1.48	246.00	0.67	4.81	33.54	44.94	20.18	1.61	40.0	18.70
	6	M	57	R218H	1.39	197.05	0.76	5.76	27.52	24.43	15.16	1.99	38.9	22.70
3	7^a^	M	20	R218H	1.65	251.94	0.56	4.45	26.87	28.10	14.88	4.72	40.1	21.10
	8	M	43	R218H	1.85	235.45	0.85	5.72	28.98	28.59	15.92	4.67	42.0	11.80
	9	F	39	R218H	1.69	194.49	0.47	5.03	30.66	35.48	21.21	1.83	41.2	17.50
4	10^ac^	F	51	R218H	1.76	268.6	0.78	5.72	30.53	40.14	21.79	1.76	44.0	19.90
	11	F	75	R218H	1.58	196.9	0.51	4.31	25.67	27.31	18.94	2,46	41.5	18.20
5	12^a^	F	25	R218H	1.10	220.00	0.79	3.10	–	31.84	–	3.10	39.6	16.60
	13	M	47	R218H	1.97	224.32	0.89	5.66	–	35.12	–	2.93	42.4	22.50
6	14^a^	F	31	R218H	1.56	218.28	0.66	5.42	–	37.35	–	5.42	42.5	25.60
7	15^ac^	M	26	R218H	2.42	252.27	0.99	5.06	–	30.11	–	1.87	43.2	24.40
8	16^acd^	M	66	R218H	1.85	220.65	0.55	5.65	–	38.67	–	2.41	43.0	17.20
Reference					1.34-2.73	78.38-157.40	0.16-0.95	0.56-5.91	12.3-23.0	7.9-18.4	9.01-19.05	0.56-5.91	35-55	13.00-39.00

a probands;

b values obtained with fourfold dilution of the calibration solution;

c patients previously treated with anti-thyroid drugs;

d patients concurrent with atrial fibrillation.

### 3.2 *ALB* gene mutation analysis

**Figure 1:**
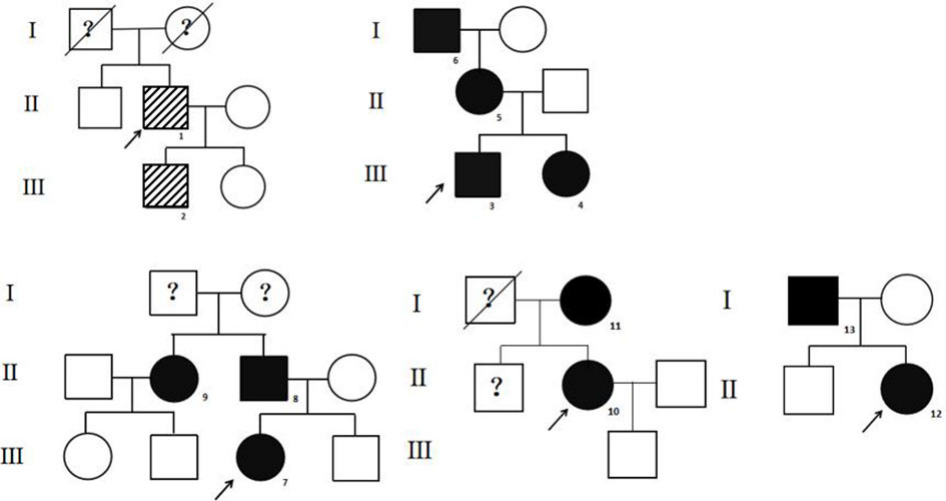
The pedigrees of the top five families. The stripe icon indicates the FDH patient with the R218S *ALB* mutation, and the black icon indicates the FDH patient with the R218H *ALB* mutation. The arrow mark indicates the proband. The question mark indicates that *ALB* mutation analysis of the individual was not performed.

The high-throughput sequencing showed patient 1 from pedigree 1 carried the heterozygous missense mutation at exon 7 of the *ALB* gene (c.724C>A; i.e., p.R218S). The patients from pedigrees 2 to 8 carried another heterozygous missense mutation at exon 7 of the *ALB* gene (c.725G>A; i.e., p.R218H; **Table 1**). There were no abnormal variants in other target genes in the panel. Notably, no subject carried pathogenic *THRβ* variants or VUS. The Sanger sequencing showed eight relatives that carried the same *ALB* gene mutations as the probands. The above two *ALB* mutation types have been directly associated with FDH and predicted to be pathogenic according to ACMG guidelines. The pedigrees of the top five families are shown in **Figure 1**. Unfortunately, family members of the other probands could not come to the hospital for evaluation for subjective or objective reasons.

### 3.3 Results of thyroid ultrasounds and RAIU of the probands

The thyroid size and blood flow signals were normal in all the probands. Three subjects underwent RAIU. The RAIU ratios of the proband 1 were near or slightly higher than the reference, and the ratios of probands 5 and 8 were both within the reference range (**Table 2**).

**Table 2:** Thyroid ultrasounds and RAIU of the probands.

Pedigree no.	Patient no.	The anteroposterior diameters of thyroid (mm)			Blood flow signal	RAIU (%)		
		The left lobe	The right lobe	The isthmus		2h	4h	24h
1	1	16	16	3.3	normal	14.5	25.6	53.2
2	3	16	15	2.7	normal	–	–	–
3	7	15	14	3.0	normal	–	–	–
4	10	15	14	2.5	normal	–	–	–
5	12	12	11	2.0	normal	9.6	14.6	25.2
6	14	14	12	2.5	normal	–	–	–
7	15	12	13	2.8	normal	–	–	–
8	16	14	13	2.5	normal	9.1	15.2	29.9
Reference		Male ≤ 17mm; Female ≤ 15mm		≤ 4mm		7-15	12-25	20-38

RAIU, ratioactive iodine uptake.

### 3.4 Serum iodothyronine levels of patients with FDH

#### 34.1 TT3, TT4, and reverse T3 (rT3) levels of the patients

The parameters of thyroid function (TT3, TT4, rT3, FT3, FT4, and TSH), serum albumin levels, and serum TBG levels are shown in **Table 1**. The calculated TT4/ULN ratios of the two patients from pedigree 1, who carried the R218S *ALB* mutation, were 9.74 and 8.05, respectively. The TT3/ULN ratios were 0.68 and 1.28, respectively. The rT3/ULN ratios were slightly elevated at 1.20 and 1.39. The TT4/ULN, TT3/ULN, and rT3/ULN ratios of the patients with the R218H *ALB* mutation were 1.44 ± 0.15, 0.65 ± 0.14, and 0.77 ± 0.18, respectively (**Table 3**). For all affected individuals, the TSH levels were 2.90 ± 1.21 μIU/ml, which were within the reference range. In addition, the serum albumin levels were 41.60 ± 1.48 g/L, and the TBG levels were 19.92 ± 3.36 μg/ml (**Table 1**).

**Table 3:** Ratio of thyroid hormones concentration to the ULN.

	Genotype	TT4/ULN	TT3/ULN	rT3/ULN
Patients from our center	R218S	9.74^a^	0.68^a^	1.20^a^
		8.05^b^	1.28^b^	1.39^b^
	R218H	1.44 ± 0.15	0.65 ± 0.14	0.77 ± 0.18
Patients from literature	R218S	11.70 ± 1.33	0.39 ± 0.11	–
	R218H	1.53 ± 0.31	1.53 ± 0.31	0.66^c^

a patient 1 from pedigree 1;

b patient 2 from pedigree 1;

c only one patient from literature having serum rT3 concentration measured (rT3/ULN was 0.66); ULN, upper limit of normal.

#### 34.2 Serum FT4 measured using chemoluminescence immunoassays with the Beckman, Roche, and Abbott platforms

**Figure 2:**
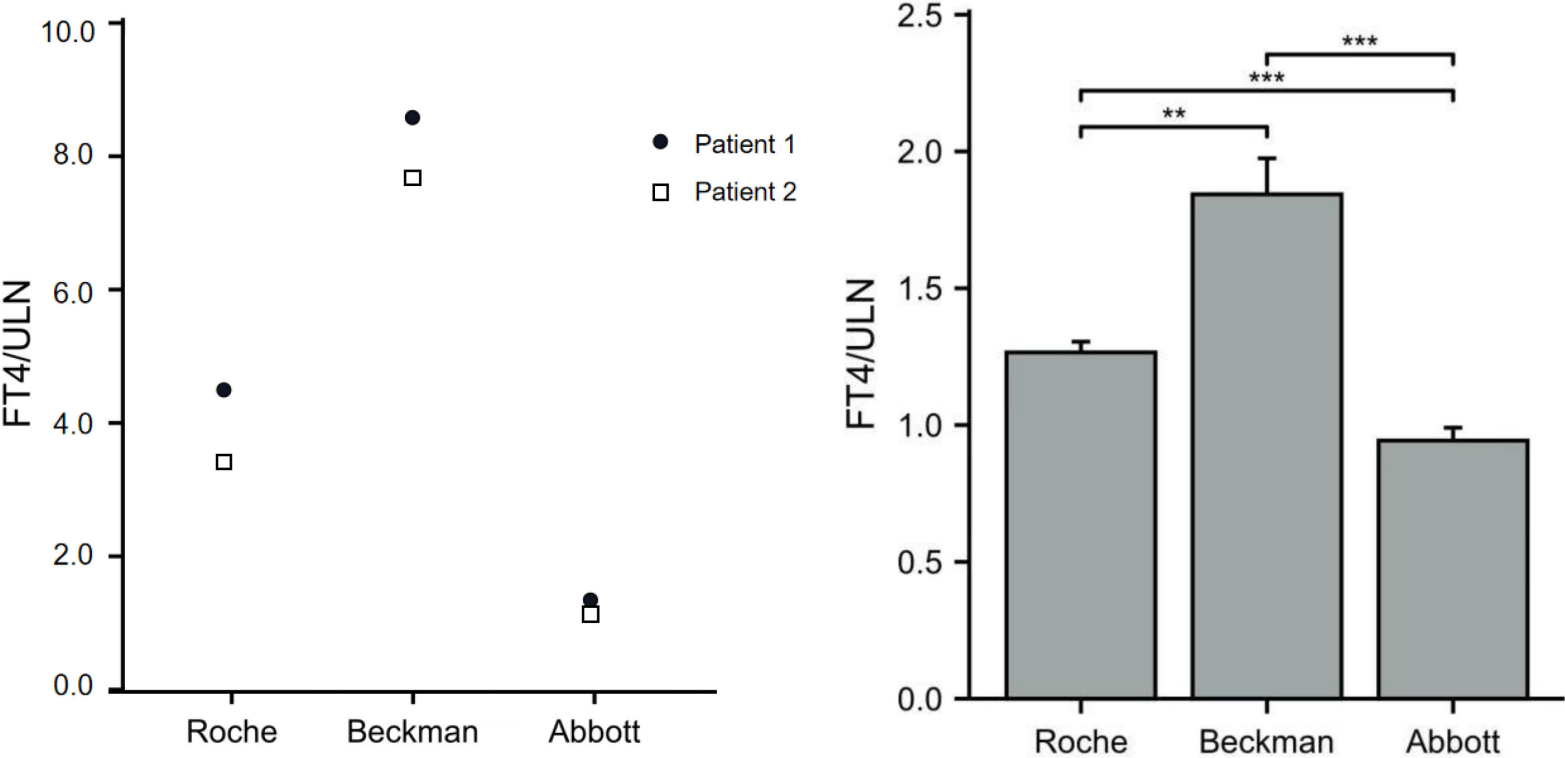
Ratios of FT4 to ULN (FT4/ULN) in FDH patients based on different assay methods. The FT4/ULN ratios of patients with the R218S mutation from pedigree 1 obtained using the three assays are shown in panel **(A)** The FT4/ULN ratios of patients with R218H mutation from pedigrees 2 to 5 were compared among the three platforms using paired samples *t*-test and results are shown in panel **(B)** Note: ^**^ *P* < 0.01, and ^***^ *P* < 0.001.

The FT4 levels of the 11 patients from pedigrees 1 to 5 were detected using chemoluminescence immunoassays with the Beckman, Roche, and Abbott platforms at the same time. FT4 measurements obtained with the three platforms were all above ULN. In patients with the R218S *ALB* mutation, the FT4/ULN ratios of patient 1 obtained from the Roche, Beckman, and Abbott platforms were 4.32, 8.64, and 1.38, respectively. The FT4/ULN ratios of patient 2 were 3.42, 7.75, and 1.21 respectively. In patients with the R218H *ALB* mutation, the FT4/ULN ratios obtained from the three platforms were significantly different. The rank order of deviation in measured *versus* reference FT4 values obtained using the three different platforms (lowest to highest) was Abbott < Roche < Beckman (all *P* < 0.05). These results are shown in **Figures 2A, B**.

### 3.5 Clinical features of the FDH patients of Chinese ethnicity from the literature

A total of 32 FDH patients of Chinese ethnicity were identified from the literature and consisted of 16 men (50%) and 16 (15%) women from nine unrelated families: Eight families carried the R218H *ALB* mutation (21 patients) and one family carried the R218S *ALB* mutation (11 patients) (4–11). Among the nine probands, 66.7% of patients (6/9) complained of symptoms such as palpitation, weight loss, and anxiety, and the other three patients (3/9) showed abnormal thyroid function tests based on routine physical examination. The age of 13 patients was recorded, and the mean age was 35.08 ± 19.23 years.

In subjects with the R218H *ALB* mutation, approximately 90% (19/21) had serum TT4 concentration measured and the TT4/ULN ratio was 1.53 ± 0.31; 52.4% of patients (11/21) had serum TT3 concentration measured and the TT3/ULN ratio was 1.49 ± 0.91; 4.7% of patients (1/21) had serum rT3 concentration measured and the rT3/ULN ratio was 0.66. In subjects with the R218S *ALB* mutation, all patients (11/11) had serum TT4 concentration measured and all values were > 386.0 nmol/L; 45.5% of the blood samples (5/11) underwent dilution tests and the TT4/ULN ratio was 11.70 ± 1.33. In addition, 90.9% of patients (10/11) had TT3 measured, and the TT3/ULN ratio was 0.39 ± 0.11 (**Table 3**).

## 4 Discussion

Euthyroid hyperthyroxinemia is a major characteristic of FDH and usually detected during a routine health examination (11, 12) or by the presence of vague signs and symptoms possibly associated with thyroid dysfunction such as palpitation, weight loss, tremors, and anxiety (10, 13). In the present study, approximately 50% of probands complained of persistent or paroxysmal palpitation, of which two probands were diagnosed with paroxysmal atrial fibrillation. These symptoms are similar to thyrotoxicosis and more likely to lead to misdiagnosis as hyperthyroidism. In addition, euthyroid hyperthyroxinemia needs to be distinguished from syndrome of inappropriate secretion of TSH (SITSH) due to similar elevated thyroid hormones levels and unsuppressed TSH levels (14). Apart from the usual investigations for a TSH-secreting pituitary adenoma such as pituitary MRI and serum TSHα submit, or a genetic testing for RTHβ, further biochemical testing of the patient’s thyroid status should be considered (10, 15, 16). Furthermore, FDH could mask the simultaneous presence of true thyroid disease (17–19). Clinical recognition of FDH is important primarily due to the risks of misdiagnosis and inappropriate treatment.

Several different codon mutations on *ALB* have been identified worldwide and vary based on ethnicity. As previously mentioned, R218H is the most common variant responsible for FDH, with a prevalence of 1:10.000 in Caucasians. It is also reported in Korean and Chinese populations (7, 21). R218P is the only mutation found to date in the Japanese population, mostly from Aomori prefecture (1, 13, 22). However, the R218P mutation is not unique to Japan, as it has also been found in a Caucasian family from Switzerland (23). R218S, a relatively rare mutation, was first reported in a Canadian family of Bangladeshi extraction (24), and recently found in American and Chinese populations (11, 25). R222I, with substitution of isoleucine for arginine at codon 222, a heterozygous *ALB* defect, has been detected in three African (Somali) subjects and one Eastern European (Croatian) family to date (26). These single-point mutations in exon 7 of the *ALB* gene change arginine in position 218 or 222 for another amino acid. Usually, these genetic variants have an increased affinity for thyroid hormones, especially for T4 (1, 24, 26). Another *ALB* mutation L66P, identified in a Thai kindred, results in a significantly increased binding affinity for T3 but not for T4 (27).

In the present study, only two patients from pedigree 1 carried the R218S mutation. R218S was first reported by Greenberg SM, who showed that the serum TT4 level of the patient was increased sevenfold and TT3 and rT3 were elevated approximately 1.6- and 2.4-fold, respectively (24). In a recent study, the TT4 levels of a female patient with the R218S mutation were approximately 9.3-fold higher than of her sister who did not have the mutation (25). In our study, the TT4 levels of the patients with the R218S mutation were approximately 8- to 9-fold of the ULN. The rT3 levels were less elevated by 1.20- and 1.39-fold. However, the TT3 levels were inconsistent between the father and son. Similarly, the TT4 levels were reportedly increased approximately 11.70-fold, and TT3 levels were in the normal range in a Chinese pedigree including 11 patients in whom the FDH was caused by the R218S mutation (11).

To date, R218H is the most common *ALB* mutation that causes FDH. Reportedly, TT4 is moderately increased 1.1- to 1.8-fold of the ULN, TT3 0.6- to 1.2-fold, and rT3 for 0.7- to 1.4-fold in subjects with the R218H mutation (1). In the present study, iodothyronine concentration of 14 FDH patients with the R218H mutation was measured in seven unrelated families. The TT4 levels were approximately 1.43-fold higher than the ULN, and both of TT3 and rT3 levels were within the reference range. In the FDH patients of Chinese ethnicity with the R218H mutation identified from the literature (4–11), the TT4/ULN ratio was approximately 1.2–1.8, and TT3 was approximately 0.6–2.4, which was somewhat consistent with those from our center. Regardless of different countries or ethnicities, the R218H mutation apparently causes a similar affinity of albumin for thyroxine.

It is intriguing that the two variants affecting the same original residue cause such a big difference in the biochemical spectrum. R218 and R222 and other residues in the mature protein important for T4 binding are reported in several studies. When the R218 or R222 is substituted for a smaller amino acid, the size of the T4-binding site may be increased as well as the affinity for T4 (2, 28). The Ser218 (218S) is within hydrogen bonding distance to the backbone carboxyl of Trp214; thus, R218S binds to T4 is much stronger than R218H (24). In this study, thyroid ultrasonography in eight probands showed no significant abnormalities in thyroid volume or blood flow signal. However, among the three patients who underwent RAIU, the patient with the R218S mutation appeared to have a higher uptake rate, which was near or above the ULN. Hypothetically, the thyroid gland of patients with the R218S mutation may need to synthesize more thyroid hormone to have sufficient TT4, a reserve pool of FT4, to maintain the normal serum FT4 concentration, since the affinity of albumin for T4 is significantly higher in patients with the R218S mutation than in patients with the R218H mutation.

Abnormal affinity of albumin for thyroxine can affect the total rather than free concentrations of iodothyronine in serum. However, the FT4 levels detected in FDH patients are usually elevated to varying degrees, depending on the specific assay kit used. Equilibrium dialysis, ultrafiltration methods, gel absorption methods, and liquid chromatography tandem mass spectrometry (LC-MS/MS) should provide nearly accurate FT4 results (25, 29). However, these methods may not be routinely available due to high technical support required, inconvenient operation, and high cost. In most clinical laboratories, FT4 is measured using automated immunometric assays including one- and two-step methods, resulting in falsely high FT4 values in FDH patients (30, 31). One-step methods rely on the competition of a T4 analog with the unbound T4 in a sample and can provide falsely high FT4 results due to the enhanced binding affinity to the T4 analog caused by the *ALB* gene mutation. In contrast, two-step assays typically utilize immobilized T4 antibodies to sequester a small proportion of total T4 from a diluted serum specimen without disturbing the original free to protein-bound equilibrium. After washing to remove unbound serum constituents, a labeled tracer is added to quantify unoccupied antibody-binding sites that are inversely associated with the free hormone concentration.

The chemoluminescence immunoassays testing thyroid function in our region are mainly conducted using three different platforms: Roche (one-step method), Beckman, and Abbott (two-step methods). In the present study, the FT4/ULN ratios obtained from the three platforms were compared with evaluate the accuracy of FT4 in FDH patients based on different methods or platforms. The results showed that all FT4/ULN ratios from the three platforms were > 1, indicating all three measurement methods were susceptible to interference. In addition, in our experience, the FT4/ULN ratios of FDH patients with R218H or R218S mutations were lowest in the Abbott platform, indicating the FT4 level detected with the Abbott platform is less affected than with the other two platforms. These results are consistent with those of a recent report in which the FT4 results from the Abbott platform were the least likely to be affected among three automated immunoassays and had the best agreement with LC-MS/MS (32). Beckman, although as a two-step method, is recorded highest FT4/ULN values; thus, interference is not a problem only exclusive to one-step assays. In some studies, T4 can reportedly be detached from albumin using chloride, anti-thyroxine antibody, or a buffer (29, 30). As others have noted previously, the FT4 assay that we found most susceptible to interference (Beckman ACCESS) uses incubation buffer with high chloride content (30). To test the effect of chloride, some researchers have used equilibrium dialysis (33) by increasing the concentration of chloride ion in the assay buffer, resulting in increased FT4 levels. Therefore, the chloride ion inhibits serum T4 binding more in patients with FDH than in normal subjects, possibly due to higher amounts of T4 bound to the mutant albumin; thus, assay buffers containing the chloride ion may cause the incorrect measurements of FT4 levels (33). However, the chloride ion is not the only interference factor leading to false FT4 measurements. Serena Khoo et al. found that increased chloride in the assay buffer significantly affected FT4 results obtained from the Ortho VITRO, but minimally affected results obtained from the Roche ELECSYS or Fyjirebio LUMIPULSE, indicating that other proprietary constituents of buffers are also responsible for assay interference in different methods (30).

Accordingly, when a patient presents with high T4 and normal TSH levels but without symptoms of thyrotoxicosis, FT4 should be remeasured using different methods or platforms. Subsequently, if different FT4 measurements are obtained, a diagnosis of FDH could be suspected.

In summary, this study was the first and largest case series of FDH caused by *ALB* mutation in the Chinese population. Two *ALB* mutations, R218S and R218H, were identified, and the latter may be a high-frequency mutation in Chinese patients with FDH. Different *ALB* mutations lead to variable affinities of serum albumin for serum iodothyronine, leading to different levels of serum iodothyronine, which appears similar to those from other countries or ethnicities. Furthermore, although the common chemoluminescence immunoassays used to detect thyroid function can produce false FT4 results in FDH patients, different levels of increased FT4 may indicate a possible diagnosis of FDH, which is important to prevent unnecessary and potentially harmful therapy. However, several limitations should be considered when interpreting our findings. First, the true values of FT4 were not detected, because the assay methods available were limited. Furthermore, due to small sample size and inadequate pedigree investigations in the present study, future studies should be conducted with a greater number of cases.

## Data availability statement

The original contributions presented in the study are included in the article/**Supplementary Material**. Further inquiries can be directed to the corresponding authors.

## Ethics statement

The studies involving human participants were reviewed and approved by The Ethics Committee of the First Affiliated Hospital of Zhengzhou University. Written informed consent to participate in this study was provided by the participants’ legal guardian/next of kin.

## Author contributions

SW and YZHAO. Study conduct: LZ, YZHOU, and FH. Data collection: LZ, XH, and GM. Data interpretation: LZ, YZHOU, FH, YZHAO, and SW. Drafting manuscript: LZ. Revising manuscript content: LZ, YZHOU, FH, and SW. Approving final version of the manuscript: LZ, YZHAO, and SW. All authors contributed to the article and approved the submitted version.
