# Supplementary material for: Clinical characteristics of familial dysalbuminemic hyperthyroxinemia in Chinese patients and comparison of free thyroxine in three immunoassay methods
Source: Front Endocrinol (Lausanne). 2023 Feb 14;14:1102777. doi: 10.3389/fendo.2023.1102777 (PMC9971560; doi:10.3389/fendo.2023.1102777)
Supplement: Supplementary file 1 [file Table_1.docx]

Supplementary Table 1 Target genes associated with thyroid diseases

| The target genes | | | | | | | |
| --- | --- | --- | --- | --- | --- | --- | --- |
| ABCA1 | CLEC7A | FOXE1 | IYD | NTRK1 | SDHD | THRB | VDR |
| AIRE | CYP27B1 | FOXP3 | MEN1 | PAX8 | SECISBP2 | TPO | ZFAT |
| ALB | CYP2R1 | GATA3 | MINPP1 | PCM1 | SLC16A2 | TRH |  |
| APOA1 | DICER1 | GCM2 | MYH8 | PHEX | SLC26A4 | TRIM24 |  |
| BCAM | DMP1 | GLIS3 | NCOA4 | PRKARIA | SLC34A3 | TRIM33 |  |
| CACNAIS | DUOX2 | GNAS | NDUFA13 | PTEN | SLC5A5 | TSHB |  |
| CASR | DUOXA2 | GOLGA5 | NKX2-1 | PTH | TBCE | TSHR |  |
| CDC73 | ENPP1 | HRAS | NKX2-5 | RET | TG | TTR |  |
| CLCN5 | FGF23 | IGSF1 | NRAS | SDHB | THRA | UBR1 |  |
